# Both higher fitness level and higher current physical activity level may be required for intramyocellular lipid accumulation in non-athlete men

**DOI:** 10.1038/s41598-020-61080-5

**Published:** 2020-03-05

**Authors:** Nozomu Yamasaki, Yoshifumi Tamura, Kageumi Takeno, Saori Kakehi, Yuki Someya, Takashi Funayama, Yasuhiko Furukawa, Hideyoshi Kaga, Ruriko Suzuki, Daisuke Sugimoto, Satoshi Kadowaki, Motonori Sato, Takashi Nakagata, Miho Nishitani-Yokoyama, Kazunori Shimada, Hiroyuki Daida, Shigeki Aoki, Hiroaki Satoh, Ryuzo Kawamori, Hirotaka Watada

**Affiliations:** 1 Department of Metabolism & Endocrinology, Juntendo University Graduate School of Medicine, Tokyo, Japan; 2 Sportology Center, Juntendo University Graduate School of Medicine, Tokyo, Japan; 3 Department of Cardiology, Juntendo University Graduate School of Medicine, Tokyo, Japan; 4 Department of Radiology, Juntendo University Graduate School of Medicine, Tokyo, Japan; 5 Center for Identification of Diabetic Therapeutic Targets, Juntendo University Graduate School of Medicine, Tokyo, Japan; 6 Center for Molecular Diabetology, Juntendo University Graduate School of Medicine, Tokyo, Japan

**Keywords:** Pre-diabetes, Lifestyle modification

## Abstract

Accumulation of intramyocellular lipid (IMCL) is observed in individuals with insulin resistance as well as insulin-sensitive endurance athletes with high peak oxygen consumption (VO_2_peak), which is called the athlete’s paradox. It remains unclear whether non-athletes with higher fitness levels have IMCL accumulation and higher insulin sensitivity in general. In this study, we investigated the association between IMCL accumulation and muscle insulin sensitivity (M-IS) in subjects with high or low VO_2_peak. We studied 61 nonobese (BMI, 23 to 25 kg/m^2^), non-athlete Japanese men. We divided the subjects into four groups based on the median value of VO_2_peak and IMCL in the soleus muscle. We evaluated M-IS using a two-step hyperinsulinemic-euglycemic clamp. Among subjects with higher VO_2_peak (n = 32), half of those (n = 16) had lower IMCL levels. Both High-VO_2_peak groups had higher M-IS than the Low-VO_2_peak groups. On the other hand, M-IS was comparable between the High-VO_2_peak/High-IMCL and High-VO_2_peak/Low-IMCL groups, whereas the High-VO_2_peak/High-IMCL group had IMCL levels that were twice as high as those in the High-VO_2_peak/Low-IMCL group. On the other hand, the High-VO_2_peak/High-IMCL group had significantly higher physical activity levels (approximately 1.8-fold) than the other three groups. In conclusion, in nonobese, non-athlete Japanese men, subjects with higher VO_2_peak and higher IMCL had higher physical activity levels. IMCL accumulation is not associated with insulin resistance in individuals with higher or lower fitness levels.

## Introduction

Insulin resistance is regarded as an important pathogenesis of lifestyle-related diseases such as metabolic syndrome and type 2 diabetes. Although the exact mechanism of insulin resistance is not fully understood, previous studies have suggested that intramyocellular lipid (IMCL) accumulation is associated with insulin resistance in muscle^1–5^. On the other hand, several studies have also shown that endurance athletes with high maximum oxygen uptake, which corresponds to a high fitness level, have high levels of IMCL accumulation despite their supernormal insulin sensitivity. This phenomenon is known as the athlete’s paradox^6^. A similar phenomenon has also been observed in non-athletes^6–9^. Indeed, we found that some individuals in the non-athlete cohort with higher fitness levels have IMCL accumulation and are insulin sensitive^8^. In addition, we previously failed to show a significant correlation between IMCL and muscle insulin sensitivity in nonobese, non-athlete Japanese men^10^. We speculated that one reason for this result could be due to some subjects having a high fitness level, substantial IMCL accumulation, and high muscle insulin sensitivity. However, it remains unclear whether non-athletes with higher fitness levels and high levels of IMCL accumulation have high insulin sensitivity. It is also unclear whether non-athletes with higher fitness levels have IMCL accumulation and higher insulin sensitivity in general. Finally, it remains unclear whether IMCL accumulation is associated with muscle insulin sensitivity in individuals with lower peak oxygen consumption (VO_2_peak).

In this context, we compared the characteristics of nonobese, non-athlete Japanese men with higher fitness levels with low versus high levels of IMCL, including insulin sensitivity and physical activity level. We also addressed the association between IMCL level and insulin sensitivity in subjects with lower fitness levels in the cohort.

## Results

**Figure 1:**
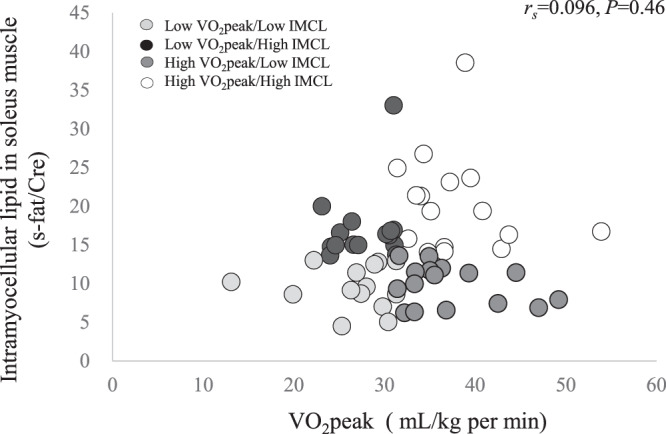
Relationship between levels of intramyocellular lipid (IMCL) in the soleus muscle and peak oxygen consumption (VO_2_peak). Subjects were divided into four groups based on the median value of VO_2_ peak (31.4 mL/kg/min) and the IMCL (13.6 s-fat/Cre).

The study subjects were 61 non-athlete Japanese men with BMI between 23 and 25 kg/m^2^. Overall, IMCL was not significantly correlated with VO_2_peak (rs = 0.096, *P* = 0.46) (Fig. 1). We divided subjects into four groups by the median value of VO_2_ peak (31.4 mL/kg/min) and IMCL in soleus muscle (SOL) (13.6 s-fat/Cre) (Fig. 1, Table 1). As shown in Table 1, the distribution of IMCL in tibialis anterior muscle (TA) among the 4 groups was similar to that of IMCL in SOL. In addition, total energy intake and dietary composition were comparable among the groups. Compared with the Low-VO_2_peak/High-IMCL group, muscle insulin sensitivity and adiponectin levels were significantly higher in the High-VO_2_peak/High-IMCL and High-VO_2_peak/Low-IMCL groups. Similarly, adipose tissue insulin sensitivity (ATIS) (%free fatty acid (FFA) suppression/insulin during the first step) was relatively lower in the Low-VO_2_peak groups. In subjects with higher VO_2_peak, half had lower IMCL levels, similar to IMCL levels in subjects in the Low-VO_2_peak/Low-IMCL group. In addition, whereas the High-VO_2_peak/High-IMCL group had IMCL levels that were 2-fold higher than levels in the High-VO_2_peak/Low-IMCL group, muscle insulin sensitivity was comparable between these two groups. On the other hand, the High-VO_2_peak/High-IMCL group had physical activity levels that were approximately 1.8 times higher than levels in the other three groups. In terms of muscle insulin sensitivity in the groups with lower VO_2_peak, although the Low-VO_2_peak/High-IMCL group had IMCL levels that were twice as high as levels in the Low-VO_2_peak/Low-IMCL group, muscle insulin sensitivity was comparable between these two groups. Trends in insulin sensitivity levels in each group were similar when we stratified subjects by VO_2_peak and IMCL level in TA (Table 2), while we observed small differences of statistical significance between the groups.

**Table 1:** Clinical characteristics of the study subjects divided based on median values of VO_2_peak and IMCL in soleus muscle.

	Low VO_2_peak Low IMCL (n = 14)	Low VO_2_peak High IMCL (n = 15)	High VO_2_peak Low IMCL (n = 16)	High VO_2_peak High IMCL (n = 16)	P*
IMCL in soleus muscle (S-fat/creatine)	9.6 ± 2.8	17.1 ± 4.7	9.8 ± 2.6	20.3 ± 6.4	
VO_2_peak (mL/kg/min)	26.4 ± 5.1	27.8 ± 3.1	37.3 ± 5.6	37.8 ± 5.6	
Age (years)	44.9 ± 4.8	42.7 ± 5.1	**39.5 **±** 4.4**^**†**^	42.1 ± 5.0	**0.02**
Body mass index (kg/m^2^)	24.2 ± 0.5	24.0 ± 0.6	24.1 ± 0.4	23.8 ± 0.6	0.36
Total body fat content (%)	22.5 ± 2.3	23.4 ± 4.3	22.1 ± 5.2	20.6 ± 4.1	0.32
Abdominal visceral fat area (cm^2^)	99.2 ± 28.2	105.6 ± 31.5	79.6 ± 22.4	90.6 ± 36.8	0.05
Abdominal subcutaneous fat area (cm^2^)	128.7 ± 27.4	123.1 ± 32.0	124.4 ± 41.6	122.5 ± 51.2	0.98
Intrahepatic lipid (%)	3.1 ± 4.7	5.8 ± 5.8	1.4 ± 1.5	3.3 ± 3.7	0.14
IMCL in tibialis anterior muscle (S-fat/creatine)	2.4 ± 1.0	3.4 ± 1.5	2.5 ± 0.9	3.6 ± 1.5^**†**^	**0.04**
Total energy intake (kcal/day)	2047.0 ± 506.1	2062.5 ± 426.7	2099.2 ± 412.1	2274.8 ± 780.1	0.65
Carbohydrate intake (kcal/day)	1159.8 ± 326.7	1133.0 ± 279.5	1184.9 ± 301.4	1249.3 ± 500.2	0.95
Protein intake (kcal/day)	307.1 ± 98.1	312.4 ± 75.4	319.6 ± 51.6	338.6 ± 119.9	0.88
Fat intake (kcal/day)	580.1 ± 196.7	617.0 ± 161.0	594.8 ± 139.2	686.9 ± 301.3	0.75
Physical activity (METs·h/day)	2.59 ± 1.60	2.48 ± 1.68	2.73 ± 1.77	**4.73 **±** 2.31**^**†‡**§^	**0.02**
Fasting plasma glucose (mg/dL)	96.1 ± 8.1	95.5 ± 9.5	93.3 ± 6.1	93.5 ± 7.7	0.70
Fasting serum insulin (μU/mL)	5.9 ± 2.7	6.9 ± 3.2	4.7 ± 2.2	5.2 ± 3.1	0.12
Free fatty acids (μEq/L)	427.9 ± 113.0	401.2 ± 113.8	377.1 ± 87.6	348.1 ± 110.9	0.22
Triglycerides (mg/dL)	134.4 ± 86.6	186.1 ± 114.0	101.9 ± 98.5	104.5 ± 48.2	**0.02**
High density lipoprotein cholesterol (mg/dL)	55.4 ± 12.1	53.7 ± 16.5	57.3 ± 14.5	63.8 ± 14.2	0.09
Total adiponectin (μg/mL)	3.5 ± 1.3	2.7 ± 1.1	**4.5 **±** 1.8**^**‡**^	**4.8 **±** 2.0**^**‡**^	**<0.01**
Log (C-reactive protein) (ng/mL)	2.51 ± 0.34	2.69 ± 0.47	2.49 ± 0.47	**2.19 **±** 0.27**^**‡**^	**0.01**
%reduction in EGP/SS_SI_ at first step (%/μU·mL^−1^)	3.3 ± 1.1	3.1 ± 0.9	3.5 ± 1.1	3.8 ± 1.0	0.29
Rd/SS_SI_ during the second step (mg/kg fat free mass·min^−^¹/μU·mL^−1^)	0.18 ± 0.08	0.15 ± 0.05	**0.24 **±** 0.08**^**‡**^	**0.25 **±** 0.08**^**‡**^	**<0.01**
%FFA suppression/insulin during the first step (%/μU·mL^−1^)	3.6 ± 1.4	3.6 ± 0.9	4.5 ± 1.3	4.6 ± 0.8	**0.02**

Data are expressed as means ± SD. *P value for one-way analysis of variance or the Kruskal-Wallis test. ^†,‡,§^P < 0.05 for the Tukey-Kramer or Games-Howell test; ^†^vs. Low-VO2peak/Low-IMCL, ^‡^vs. Low-VO2 peak/High-IMCL, ^§^vs. High-VO2 peak/Low-IMCL.

EGP, endogenous glucose production; FFA, free fat acid; IMCL, intramyocellular lipid; METs, metabolic equivalents; Rd, rate of disappearance; S-fat, methylene signal intensity; SS_SI_, steady-state serum insulin; VO_2_peak, peak oxygen consumption.

**Table 2:** Insulin sensitivity of the study subjects divided based on median values of VO_2_peak and IMCL in tibialis anterior muscle.

	Low VO_2_peak Low IMCL (n = 16)	Low VO_2_peak High IMCL (n = 13)	High VO_2_peak Low IMCL (n = 14)	High VO_2_peak High IMCL (n = 17)	P*
IMCL in tibialis anterior muscle (S-fat/creatine)	1.9 ± 0.6	4.0 ± 1.1	1.9 ± 0.5	4.0 ± 1.1	
VO_2_peak (mL/kg/min)	26.8 ± 5.0	27.6 ± 3.0	38.6 ± 7.0	36.9 ± 4.1	
%reduction in EGP/SS_SI_ at first step (%/μU·mL^−^¹)	3.2 ± 0.9	3.1 ± 1.1	3.5 ± 1.0	3.7 ± 1.1	0.43
Rd/SS_SI_ during the second step (mg/kg fat free mass·min^−^¹/μU·mL^−^¹)	0.18 ± 0.07	0.15 ± 0.07	**0.25 **±** 0.09**^**†‡**^	0.22 ± 0.06	**<0.01**
%FFA suppression/insulin during the first step (%/μU·mL^−^¹)	3.7 ± 1.1	3.5 ± 1.3	4.4 ± 1.3	4.5 ± 0.9	**0.03**

Data are expressed as means ± SD. ^*^P value for one-way analysis of variance or the Kruskal-Wallis test. ^†,‡^*P* < 0.05 for the Tukey-Kramer or Games-Howell test; ^†^vs. Low-VO_2_peak/Low-IMCL, ^‡^vs. Low-VO_2_ peak/High-IMCL.

## Discussion

It remains unclear whether non-athlete subjects with higher fitness levels in general had IMCL accumulation and higher insulin sensitivity. This study showed that compared with the Low-VO_2_peak/High-IMCL group, muscle insulin sensitivity and adiponectin levels were significantly higher in the two groups with higher VO_2_peak. Half of subjects with higher VO_2_peak had IMCL accumulation and High-VO_2_peak/High-IMCL group had significantly higher physical activity levels than the other three groups. Contrary to our hypothesis, muscle insulin sensitivity was comparable between the Low-VO_2_peak/Low-IMCL and Low-VO_2_peak/High-IMCL groups. Similarly, IMCL accumulation was not associated with insulin sensitivity in subjects with higher VO_2_peak.

The athlete’s paradox is generally defined as the phenomenon in which highly trained endurance athletes with high fitness levels have enhanced insulin sensitivity in muscle despite IMCL accumulation. Interestingly, our data showed that even if fitness level is higher, half had lower IMCL levels, similar to levels in the Low-VO_2_peak/Low-IMCL group. We found that physical activity level is significantly higher in the High-VO_2_peak/High-IMCL group than in the other three groups. A previous study demonstrated that exercise training increases intramyocellular triglyceride levels and insulin sensitivity and decreases diacylglycerol levels in obese subjects^7^. In addition, a single bout of exercise increased intramyocellular triglyceride levels during lipid infusion and decreased muscle diacylglycerol levels, possibly due to enhanced diacylglycerol acyltransferase expression^11^. We also previously showed that physical activity level is positively correlated with increased IMCL during a 3-day high-fat diet in healthy subjects with high fitness levels^12^. Taken together, both higher fitness level and higher current physical activity level may be required for IMCL accumulation in non-athletes as well as the athlete’s paradox phenomenon.

The present study showed that IMCL accumulation is not associated with insulin resistance in subjects with higher or lower fitness levels. Similarly, several studies have revealed that ectopic fat content in muscle and liver is not associated insulin resistance^6,8,13–19^. It might be possible that IMCL levels observed in the High-IMCL groups were too small to cause insulin resistance. Our previous study demonstrated that obese subjects with metabolic syndrome have muscle insulin resistance and IMCL levels in SOL and TA of 14.7 ± 6.4 (S-fat/Cre) and 3.5 ± 2.1 (S-fat/Cre), respectively^10^. Thus, as shown in Table 1, it seems that the IMCL levels observed in the High-IMCL groups have potential to cause insulin resistance. On the other hand, muscle insulin sensitivity is regulated by factors such as blood flow, capillary density, and levels of proteins related to insulin signaling and chronic inflammation^7,20–22^. Thus, it is possible that factors other than IMCL accumulation may play a larger role in regulating insulin sensitivity. In addition, IMCL levels measured with ^1^H-MRS mainly reflect triglycerides; however, candidate lipids that cause insulin resistance do not include triglycerides but rather other lipid species such as ceramide and diacylglycerol^20^. In fact, ceramide and diacylglycerol have been suggested to activate protein kinase C and inhibit insulin signal transduction, thus inducing insulin resistance^23^. These might be reasons why IMCL levels in the present study were not associated with insulin sensitivity in subgroups based on fitness level. Further analysis of muscle biopsy findings is clearly required to confirm this hypothesis.

In the present study, adiponectin levels were generally higher in the groups with higher VO_2_peak compared with lower VO_2_peak. A previous report suggested that adiponectin activates AMP kinase (AMPK) and improves insulin resistance^24^. Adiponectin has been reported to increase mitochondrial biogenesis, oxidative metabolism, and type I (oxidative) myofiber in muscle via the adiponectin receptor 1 (AdipoR1)-AMPK-peroxisome proliferator-activated receptor-γ coactivator-1α axis^25^. Administration of the adiponectin receptor agonist AdipoRon increases muscle oxidative capacity, insulin sensitivity, and endurance exercise capacity in rodents^26^. Similarly, other reports have shown that VO_2_peak, type I myofiber^7,9^ and capillary density^7^ as well as adiponectin concentration^27^ are significantly increased with exercise and changes in adiponectin levels are significantly correlated with changes in VO_2_peak^27^. In addition, type I myofibers are more sensitive to insulin despite containing more IMCL than type II fibers^23^, probably due to having higher protein levels of insulin receptor and glucose transporter-4 and higher capillary density. Thus, adiponectin levels might be associated with VO_2_peak and subjects with higher VO_2_peak and adiponectin might have more type I myofibers, which protect them against IMCL-induced insulin resistance. Further study is required to support this hypothesis.

Similar to adiponectin levels, ATIS was relatively higher in the groups with higher VO_2_peak than lower VO_2_peak. It has been suggested that reduced ATIS corresponds to inadequate suppression of FFA release from adipose tissue by insulin, and the released FFAs subsequently induce insulin resistance in muscle^28,29^. Given that adiponectin levels are lower in obese individuals^30^, reduced ATIS and adiponectin levels may both reflect adipose tissue dysfunction. Even in nonobese subjects, reduced ATIS and adiponectin levels were associated with insulin resistance and metabolic disorders^10,31^. Although the effect of VO_2_peak on ATIS remains unclear, relatively impaired adipose tissue function in the Low-VO_2_peak groups might contribute to reduced insulin sensitivity in muscle.

In conclusion, nonobese, non-athlete Japanese men with higher IMCL and fitness levels have higher insulin sensitivity and physical activity levels. This finding suggests that higher fitness level and higher current physical activity level may both be required for the athlete’s paradox phenomenon. IMCL accumulation is not associated with insulin resistance in subjects with higher or lower fitness levels. Fitness level is a better parameter for predicting insulin sensitivity, regardless of IMCL level.

## Research Design and Methods

### Study subjects

The Sportology Center Core Study was a prospective observational study involving hypothesis-driven, hypothesis-generating research on the mechanisms underlying metabolic abnormalities in non-diabetic Japanese men^10^. That study enrolled non-diabetic Japanese men with a BMI of 21 to 27.5 kg/m^2^ (≥21.0 to <27.5 kg/m^2^) who were between 30 and 50 years old. In the current study analysis, we selected 61 subjects with BMI between 23 and 25 kg/m^2^ (≥23.0 to <25.0 kg/m^2^). All participants gave written informed consent to the study, which was approved by the ethics committee of Juntendo University (No.2022042). This study was carried out in accordance with the principles outlined in the Declaration of Helsinki.

### Study design

The design of the Sportology Center Core Study was described previously^10^. Briefly, all participants were included through the screening session participated in 3-visit examination for baseline evaluation. At the first or second visit, each subject underwent oral glucose tolerance test or peak oxygen uptake test after an overnight fast. Diet history was evaluated using a brief self-administered questionnaire^32^. Physical activity levels before this study were evaluated using the International Physical Activity Questionnaire (IPAQ)^33^. Voluntary exercise was prohibited for 10 days before the third visit. The mean daily physical activity (e.g. walking) level was estimated over 7 days with an accelerometer (Lifecorder; Suzuken, Nagoya, Japan). Subsequently, each subject was asked to maintain their daily physical activity level at his mean daily physical activity level ± 10% during the last 3 days before the third visit. Each participant was asked to consume a standard weight-maintaining diet for the 3 days preceding the clamp study, which was performed during the third visit.

On the third visit, we evaluated fat distribution and insulin sensitivity after an overnight fast. Briefly, we measured IMCL and intrahepatic lipid (IHL) levels using ^1^H-magnetic resonance spectroscopy (MRS) and estimated abdominal visceral fat area and subcutaneous fat area (SFA) using magnetic resonance imaging (MRI). Total body fat content and fat-free mass (FFM) were measured by the bioimpedance method (InBody; Biospace, Tokyo)^34^. Then, the participants underwent euglycemic hyperinsulinemic clamp to measure insulin sensitivity in muscle and liver.

### Measuremnet of cardiorespiratory fitness

All participants underwent an incremental cycling test (Corival 400, Lobe B.V., Groningen, Netherlands) using an expiratory gas analyzer (Vmax-295, SensorMedics Co., Yorba Linda, CA) to measure VO2peak. After a 3-min rest period, the subject cycled for 3-min warm-up at 40 W. This was followed by ramp loading (15 W/min) until subjective exhaustion, as described previously^35^.

### Intra-abdominal and subcutaneous fat

Intra-abdominal and subcutaneous fat areas were measured with MRI as described previously^36^. Briefly, T1-weighted trans-axial scans were obtained intra-abdominal and subcutaneous fat areas at the fourth and fifth lumbar interspaces were measured as described previously by using specific software (AZE Virtual Place, Tokyo, Japan)^36^.

### ^1^H-MRS

IMCL of the TA and SOL and IHL of segment 6 in the liver were measured using ^1^H-MRS (VISART EX V4.40, Toshiba, Tokyo)^36,37^. After ^1^H-MRS measurements, IMCL was quantified using methylene signal intensity (S-fat) with the creatine signal (Cre) as the reference and was calculated as a ratio S-fat/Cre. IHL was quantified by S-fat and H_2_O as the internal reference, and calculated as a percentage of H_2_O + S-fat [S-fat x 100 / (H_2_O + S-fat)]^36,37^.

### Euglycemic hyperinsulinemic glucose clamp

A two-step euglycemic hyperinsulinemic glucose clamp study was performed with an artificial endocrine pancreas (STG 22; Nikkiso, Shizuoka, Japan)^10^. Briefly, after securing an intravenous cannula in the forearm, a bolus dose [200 mg/m^2^ body surface area (BSA)] of [6,6-^2^H_2_]glucose (Cambridge Isotope Laboratories, Tewksbury, MA) was injected intravenously, followed by constant infusion of 2 mg/m^2^ BSA per min for 3-h (−180 to 0 min) to measure fasting endogenous glucose production (EGP)^38^. This was followed by primed insulin infusion (40 mU/m^2^ per min followed by 20 mU/m^2^ per min, each lasting 5 min) and continuous insulin infusion at 10 mU/m2 per min for 3 h (first step) (0 to 180 min). In the second step of the clamp, after a priming insulin infusion (80 mU/m^2^ per min followed by 40 mU/m^2^ per min, each lasted 5 min), insulin was infused continuously at 20 mU/m^2^ per min for 3 h (180 to 360 min). We used a warming blanket for arterialization of hand vein. Plasma glucose level in arterialized blood was maintained at ~95 mg/dl by a variable 20% glucose infusion containing ~2.5% [6,6-^2^H_2_]glucose. Blood samples were obtained for biochemical analysis at 10 min intervals during the last 30 min of the steady state periods of the first and second steps of the clamp. Enrichment of [6,6-^2^H_2_]glucose in plasma was measured by high-performance liquid chromatography (LTQ-XL-Orbitrap mass spectrometer, Thermo Scientific, CA) as described previously^10^. A steady state equation was used to calculate the rates of EGP and rate of disappearance (Rd) at each step^39^. EGP and Rd were normalized by BSA and FFM, respectively^10^. We divided % reduction of EGP at the first step by the steady state serum insulin (SS_SI_) and used it as an index of hepatic insulin sensitivity^40^. Similarly, Rd of glucose at the second step was divided by SS_SI_ and used it as an index of muscle insulin sensitivity^41^. Percent reduction of FFA at the first step was calculated by basal and nadir FFA concentrations during the last hour of glucose clamp during the first step and adjusted by the insulin concentration, which was used as an index of adipose tissue insulin sensitivity^31^.

### Statistical analysis

Data are presented as means ± SD. The relationship between IMCL and VO_2_peak was assessed using Spearman’s correlation coefficient. For group comparisons, we divided subjects into four groups based on the median value of VO_2_ peak (31.4 mL/kg/min) and IMCL in SOL (13.6 s-fat/Cre): Low-VO_2_peak/Low-IMCL group (n = 14), High-VO_2_peak/Low-IMCL group (n = 16), Low-VO_2_peak/High-IMCL group (n = 15), and High-VO_2_peak/High-IMCL group (n = 16) (Fig. 1 and Table 1). It has been suggested that IMCL in TA is more easily increased by 3-day fat loading than IMCL in SOL^12,42,43^. Thus, we mainly used the IMCL in SOL data to categorize subjects in order to reduce the effect of dietary fat intake on IMCL. In addition, we preliminary divided subjects based on median value of VO_2_ peak and IMCL in TA (2.8 s-fat/Cre), and compared insulin sensitivity. Data were compared among the groups with one-way analysis of variance or the Kruskal-Wallis test. Groups were then compared using a Tukey-Kramer or Games-Howell post hoc test. All statistical tests were two-sided with a 5% significance level.
