# Author Correction: Both higher fitness level and higher current physical activity level may be required for intramyocellular lipid accumulation in non-athlete men

**DOI:** 10.1038/s41598-021-86892-x

**Published:** 2021-03-25

**Authors:** Nozomu Yamasaki, Yoshifumi Tamura, Kageumi Takeno, Saori Kakehi, Yuki Someya, Takashi Funayama, Yasuhiko Furukawa, Hideyoshi Kaga, Ruriko Suzuki, Daisuke Sugimoto, Satoshi Kadowaki, Motonori Sato, Takashi Nakagata, Miho Nishitani-Yokoyama, Kazunori Shimada, Hiroyuki Daida, Shigeki Aoki, Hiroaki Satoh, Ryuzo Kawamori, Hirotaka Watada

**Affiliations:** 1 Department of Metabolism and Endocrinology, Juntendo University Graduate School of Medicine, Tokyo, Japan; 2 Sportology Center, Juntendo University Graduate School of Medicine, Tokyo, Japan; 3 Department of Cardiology, Juntendo University Graduate School of Medicine, Tokyo, Japan; 4 Department of Radiology, Juntendo University Graduate School of Medicine, Tokyo, Japan; 5 Center for Identification of Diabetic Therapeutic Targets, Juntendo University Graduate School of Medicine, Tokyo, Japan; 6 Center for Molecular Diabetology, Juntendo University Graduate School of Medicine, Tokyo, Japan

Correction to: *Scientific Reports* 10.1038/s41598-020-61080-5, published online 05 March 2020

The original version of this Article contained a typographical error in the spelling of the author Hiroaki Satoh, which was incorrectly given as Hiroaki Sato. This has now been corrected in the PDF and HTML versions of the Article.

